# Fungal dysbiosis facilitates inflammatory bowel disease by enhancing CD4+ T cell glutaminolysis

**DOI:** 10.3389/fcimb.2023.1140757

**Published:** 2023-04-14

**Authors:** Minhao Yu, Hui Ding, Shuai Gong, Yang Luo, Haiping Lin, Yifei Mu, Hao Li, Xiaobo Li, Ming Zhong

**Affiliations:** ^1^Department of Gastrointestinal Surgery, Renji Hospital, School of Medicine, Shanghai Jiao Tong University, Shanghai, China; ^2^Division of Gastroenterology and Hepatology, Renji Hospital, School of Medicine, Shanghai Jiao Tong University, Shanghai, China; ^3^Shanghai Institute of Digestive Disease, National Health Commission (NHC) Key Laboratory of Digestive Diseases, Shanghai, China

**Keywords:** IBD - inflammatory bowel disease, fungi - fungi interactions, glutaminolysis, CD4 T cell +, oxidative phosphorylation (OXPHOS)

## Abstract

The fungal microbiota is an important component of the complex multikingdom microbial community colonizing the mammalian gastrointestinal tract and has an important role in immune regulation. However, how fungi regulate inflammatory bowel disease (IBD) is poorly understood. This study found that intestinal fungi regulate immune responses in IBD. Antibiotic-mediated depletion of fungi facilitated the development of IBD. Fungi greatly enhanced oxidative phosphorylation (OXPHOS) by enhancing glutaminolysis. Mechanistically, we found that fungi could activate the dectin-1-Syk- NF-κB signaling pathway to promote the expression of key enzymes and transporters involved in glutaminolysis. In summary, our findings reveal that fungal interactions in the human gut could be a promising therapeutic target for IBD.

## Introduction

Inflammatory bowel disease (IBD), comprising Crohn’s disease (CD) and ulcerative colitis (UC), is a chronic and incurable disease associated with a growing burden worldwide (Kaplan, 2015; Faye et al., 2022). The most prominent feature of IBD is chronic immune-mediated intestinal inflammation, which is affected by genetic predisposition and diet diversity, antibiotic use, and socioeconomic development (Pytrus et al., 2022). The specific pathogenesis of IBD is unclear. Recently, gut microbiota emerged as a key player in the pathogenesis of IBD (Ni et al., 2017), even though the specific functions and mechanistic relationships of gut microbiota and immune cell metabolism reprogramming are poorly understood.

Previously, most microbiome studies focused on bacteria and were less centered on fungi, which are another important constituent of gut microbiota (Parfrey et al., 2011), including the human gut (Fujimura et al., 2016; Arrieta et al., 2018). Gut fungi play crucial roles in sustaining the homeostasis of humans by preventing pathogenic bacterial infections and protecting against intestinal complications (Hajjar et al., 2022). In addition, emerging evidence are revealing the roles of fungal microbiome in human pathology. Hu et al. (2022) and Ankit et al (Malik et al., 2018). revealed that fungi could promote the infiltration of MDSCs cells in CRC tumor microenvironments, resulting in CRC progression. In IBD, Harry et al. reported that the ratio of *Saccharomyces cerevisiae* decreased while that of *Candida albicans* increased significantly compared to healthy subjects (Sokol et al., 2017). In addition, the amount of *Malassezia*, another fungus, was associated with the burden of CD, promoting the progression of inflammation in the mouse models (Limon et al., 2019). However, how fungi directly affect the immune response remains unknown.

Emerging studies reported that metabolic reprogramming is greatly involved in the immune response of immune cells (Dominguez-Villar and Hafler, 2018; Tang et al., 2023). When CD8+ cells switch from antigen-specific effectors to long-lived memory cells, mitochondrial fatty acid oxidation dramatically increases to match functional demands (Pearce et al., 2009). Growing evidence indicates that the type of nutritional factors supplied are associated with the response of immune cells during pathogenesis. Glutamine, a non-essential amino acid, can easily integrate into multiple metabolic pathways, resulting in conditions essential for immune cell proliferation and cytokine production (Newsholme, 2001). Glutamine can act as a substrate for anabolic and catabolic metabolism for macromolecule biosynthesis and energy production (DeBerardinis et al., 2007). For most immune cells, the demand for glutamine is similar to or even greater than for glucose to support the expression of key lymphocyte cell surface markers and transcription factors (Curi et al., 2005a; Curi et al., 2005b). Yet, it is still unknown whether glutamine metabolism reprogramming is associated with fungi infection.

In this study, we found that the amount of fungi was significantly higher in feces derived from CD and UC patients compared to the healthy controls. Moreover, fungi depletion by terbinafine could significantly slow the progression of IBD. Fungi infection elevated oxidative phosphorylation (OXPHOS) by facilitating glutaminolysis. Mechanistically, we found that fungi could activate the dectin-1-Syk- NF-κB signaling pathway to promote the expression of key enzymes and transporters involved in glutaminolysis. In summary, these findings uncover that fungi could reprograming the CD4+ T cell glutamine metabolism to promote the progress of IBD

## Materials and methods

### Animal studies

Animal studies were performed according to the protocol approved by the Institutional Animal Care and Use Committee of Shanghai Jiaotong University School of Medicine, Renji Hospital (#2021-120). To establish a DSS-induced IBD model, 2.5% DSS (MP Biomedicals) was added to the drinking water of C57BL/6J mice for one week. Meanwhile, to establish a 2,4,6-trinitrobenzenesulfonic acid (TNBS)-induced IBD model, 100 ug/g TNBS in 50% ethanol solution was gradually injected into the colon of C57BL/6J mice. IL-10 KO mice were purchased from Wukong biotechnology company. The weight of the mice were measured every day. At the endpoint of the studies, mice were sacrificed, and colon and CD4+ T cells were collected for further experiments.

Human samples

Colonic and CD4+ T cells of healthy donors, UC (including stable and activated status) and CD (including stable and activated status) used in this study were obtained from the Biological Sample Bank of Renji Hospital. This study was approved by the Shanghai Jiaotong University School of Medicine, Renji Hospital Ethics Committee (#KY2021-120-B).

### *In situ* hybridization

In situ hybridization assay was used to detect the amount of fungi in colonic specimens of IBD patients, according to previous reports (Montone et al., 2011). Briefly, the colonic specimens were fixed, dewaxed, and rehydrated. Then, 18S DNA probes labeled with biotin probes were incubated with the section for one hour, with further incubation using fluorescence streptavidin. Finally, signaling was detected using a microscope.

### Histopathology and immunohistochemistry

For histopathology, the colon of IBD mice was collected and fixed after embedding in paraffin. Then, 5 μm tissue sections were stained with hematoxylin and eosin, and data were acquired using a microscope. For immunohistochemistry, the human and mice slides were de-paraffinized, and epitopes were retrieved following endogenous peroxidase blocking. Next, the tissue section was blocked with 10% BSA following primary antibody incubation: Interferon-gamma (abcam, ab231036), with 1:100 dilution; IL-17A (abcam, ab79056), with 1:100 dilution; and TNF-alpha (abcam, ab215188). Secondary antibodies were then incubated at room temperature for two hours. Finally, DAB regent was applied for signaling presentation.

The scores of histopathology and immunohistochemistry were evaluated by two blinded pathologists from Renji Hospital.

### ChIP assay

ChIP assay was conducted using a ChIP kit (abcam, ab500) following the manufacturer’s guidelines. Briefly, CD4+ T cells isolated from TNBS-induced IBD mice administrated with fungi were fixed, lysed, and sonicated. Then, the cell lysates were incubated with NF-κB p65 (CST, #8242) or immunoglobulin G (IgG). Finally, the GLS2 DNA was detected using RT-PCR.

Quantitative real-time PCR

CD4+ T cells were collected and lysed in TRIzol Plus (Takara) to extract total RNA, and 500 ng total RNA was reverse transcribed into cDNA using PrimeScript RT Master Mix (Takara). The cDNA was further mixed with SYBR Green PCR Master Mix and specific primers (Takara) to detect relative mRNA expression of specific genes.

### Cytokine measurement

The culture medium of CD4+ T cells was collected and centrifuged, and the supernatants were transferred into new tubes for enzyme-linked immunosorbent assay. Plasma levels of cytokines IFN-γ, IL-17A and TNF-α were detected using enzyme-linked immunosorbent assay (ELISA) assay kits (eBioscience) following the instructor’s guidelines.

### Gene knockdown

The siRNA was used to knock down genes. Briefly, siRNA targeting dectin-1 and dectin-3 were mixed with jetPRIME (Polyplus) for 10 min at room temperature and transferred into the CD4+ culture medium for 48 h. Cytokine expression levels were then measured by ELISA.

OCR analysis

The OCR analysis was detected using Seahorse XFe96 extracellular flux analyzer, and the experiments were conducted as previously reported (Hu et al., 2021). Briefly, human and mice CD4+ T cells were seeded into the seahorse 96-well culture plate (in triplicate) for 16 h. Before measurement, the cells were washed twice with a seahorse assay medium. Oligomycin, FCCP, and rotenone/antimycin A were added in sequence. The data were normalized with the total protein of the cells.

Statistical analysis

Data were presented as means ± SD. Data were collected from at least three independent experiments. No statistical method was used to pre-determine sample size. Two-tailed unpaired student t-test was used compare two groups, while one-way analysis of variance (ANOVA) was used to compare multiple groups. Statistical significance was set based on the P value as follows: n.s., P > 0.05; *P < 0.05, **P < 0.01, and ***P < 0.001

## Results

### Fungi increased in active IBD patients and mice

**Figure 1:**
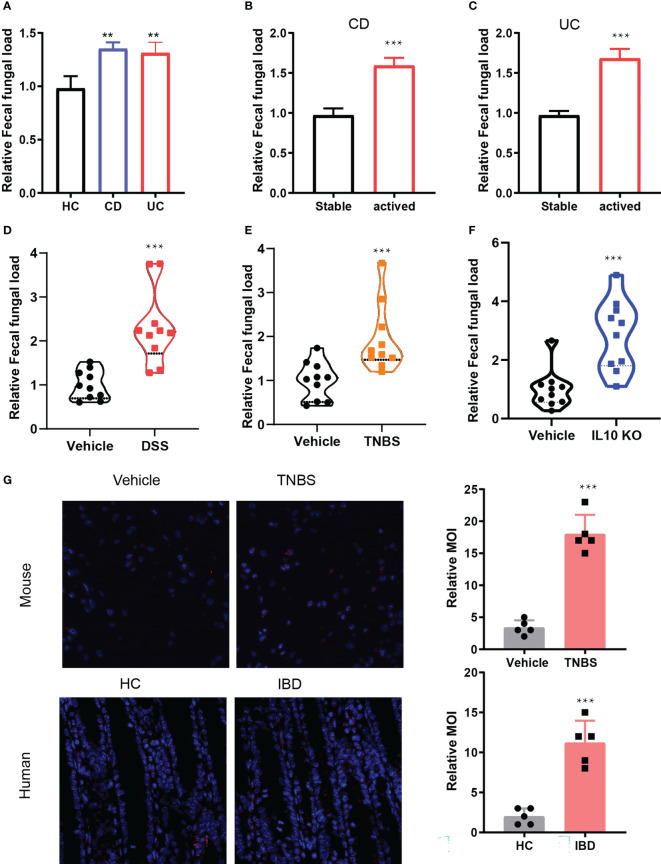
The amount of fungi increased in IBD. **(A)**. Fungal DNA in fecal samples from healthy control (HC), CD and UC patents by detecting 18S rRNA, n = 40. **(B, C)**. The amount of fungal DNA in the activate status of CD **(B)** and UC, n = 20 **(D)**. **(E–G)**: The relative fungal DNA in the control and IBD mice model including DSS induced IBD, n = 5. **(E)**, TNBS induced IBD, IL10 KO induced IBD. **(H)** *In situ* hybridization of fungi in the human and mice colon specimens. **P < 0.01, and ***P < 0.001.

Given that gut microbiota play an important role in the pathogenesis of IBD and fungi are an integral part of microbial environments, we investigated the changes in the amount of fungi in IBD. As expected, we found that the amount of fungi derived from feces of CD and UC patients was substantially more than that from healthy controls (**Figure 1A**). In addition, considering the status of IBD, we investigated whether the amount of fungi was associated with disease activities. We collected the stable and inflamed status fungi of CD and UC patients. Consistently, the amount of fungi in the activated status dramatically increased compared to the stable status (**Figures 1B, C**). To further confirmed this, we established experimental colitis models in wild-type (WT) C57BL/6 mice using TNBS and DSS and generated IL-10 KO mice. In line with the above results, the amount of fungi in the inflamed DSS-treated, TNBS-treated, and IL10 KO mice was substantially higher than in the initial state (**Figures 1D–F**). To further corroborate our preceding findings, *in situ* hybridization was performed to detect the fungi in the human and mice inflamed colon mucosa. Immunofluorescence staining results showed stronger fluorescence intensities in human and mice inflamed colon mucosa than their control counterparts (**Figure 1G**). Taken together, these findings indicate that fungi were increased in the activated status of IBD and contributed to the development of IBD.

### Fungi depletion protects against TNBS-induced colitis

**Figure 2:**
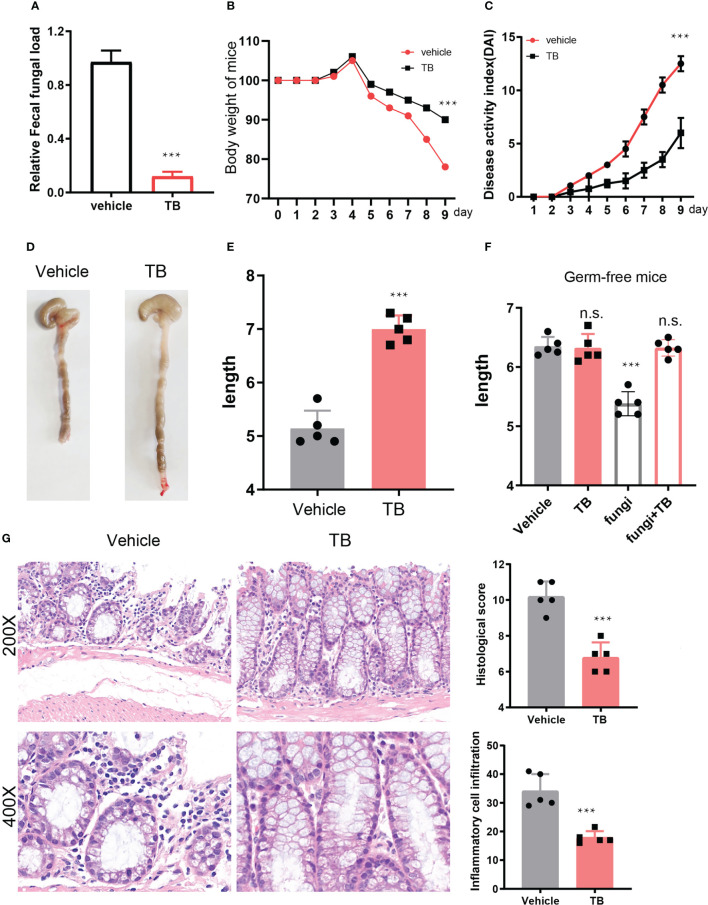
depletion fungi delayed the development of IBD. **(A)**. Fungal DNA in fecal samples from control and TB treated mice by detecting 18S RNA. **(B)**. The weight change curve of TNBS induced colitis under the treatment of terbinafine or not, n = 5. **(C)**. Disease activity index of mice IBD that treated with terbinefine or control counterpart, n = 5. **(D)**. Colon length of mice IBD with administration of terbinafine or not, n = 5. **(E)**. the statistical results of colon length in two group of mice, n = 5. **(F)**. the statistical results of colon length in germ-free mice that treated with TB, fungi or both TB and fungi. **(G)**. hematoxylin and eosin (H & E) staining of the colon sections from tebinafine treated or control group mice. ***P < 0.001 and n.s., no significance P > 0.05.

To investigate whether fungi dysbiosis affects the development of IBD, we depleted fungi in the TNBS-derived colitis mouse model. The IBD mice were injected intraperitoneally with 10mg/kg of the anti-fungi drug terbinafine (TB) every three days. As expected, the abundance of fungi dramatically decreased upon TB treatment (**Figure 2A**). The body weight change curve showed that the weight of both groups of mice decreased upon TNBS administration; however, the terbinafine treated group exhibited substantially lesser weight loss from days 4–9, with about a 12% weight difference at the end time point compared to the control group (**Figure 2B**). The disease activity index (DAI) was measured during the development of colitis. We found the control mice exhibited irregular shape stools and more severe bleeding than terbinafine-treated mice, resulting in a 45% difference in DAI between the two groups (**Figure 2C**). Furthermore, terbinafine treatment alleviated colon shortening due to the progression of colitis (**Figure 2D**). The length of the colon derived from the control group was about one centimeter shorter than that of their counterpart (**Figure 2E**). Next, we treated the germ-free mice with fungi, which promotes the IBD progression. After that, terbinefine was administrated to the fungi transferred germ-free mice. The result showed that terbinefine could reverse the progression of IBD induced by fungi. Collectively, these data suggested terbianfine exerted their function by depleting the fungi in mice but not the alteration of gut bacterial community after antifungal therapy (**Figure 2F**). Additionally, colon tissues were collected, fixed, and stained with hematoxylin and eosin (H&E). Histopathological results showed that the colon sections from the control group of mice exhibited more severe ulceration and erosion, inflammatory cell infiltration, and mucosa thickening compared with the terbinafine-treated group, resulting in higher disease scores (**Figure 2G**). In summary, these results indicate that fungi depletion could prevent the development of inflammation in the colon.

### Fungi infection promotes CD4+ T Cell immune responses in IBD patients

**Figure 3:**
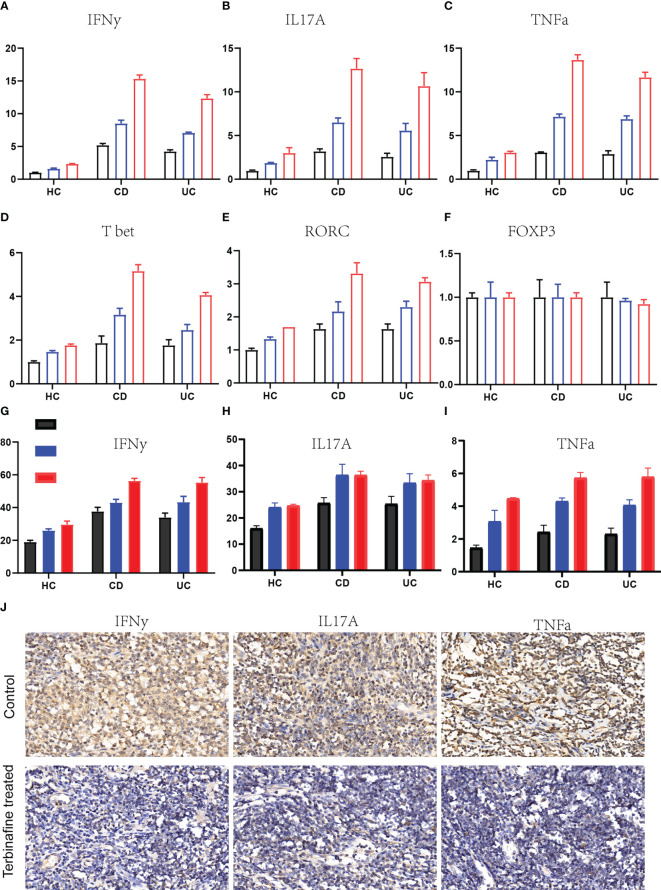
Fungi infection facilities the production of inflammation cytokines in CD4+ T cells. **(A-C)**. The relative IFNγ **(A)**, IL17A **(B)** and TNFα, n = 5. **(C)** genes expression in the CD4+ T cells drived from HC, CD and UC patients that infected with different dose of *Candida albicans*, n = 5. **(D–F)**. The expression changes of transcription factors of T-bet-Th1, n = 5. **(D)**, RORC-Th17 **(E)** and Foxp3-Treg **(F)** under fungal infection. **(G–I)**. The protein expression of IFNγ **(G)**, IL17A **(H)** and TNFα **(I)** in ther supernatant of CD4+ T cells culture medium detected by enzyme-linked immunosorbent assay, n = 5. **(J)**. The IHC staining of IFNγ, IL17A and TNFα in the colon specimens that received the terbinafine or control treatment.

Next, we aimed to elucidate the mechanisms through which fungi promote the pathogenesis of IBD. Given that CD4+ T cells are greatly involved in the initiation and development of IBD, we firstly detected whether the function of fungi in IBD depending on CD4+ T cells. The *Cd4* knock out mice that induced IBD by DSS were treated with terbinafine, the results showed that fungi depletion mediated by terbinfine could not alleviate the IBD progression, indicating that the effect of fungi relying on the CD4+ cells (**Supplementary Figure 1**). Further, we evaluated the effects of CD4+ T cell differentiation under fungi treatment. The CD4+ T cells in the peripheral blood were collected from active IBD patients and healthy controls following different doses of fungi infection. To uncover the response of CD4+ T cells, the levels of mRNA expression of typical inflammation cytokines IFN-γ, IL-17A, and TNF-α were detected in both control and fungi-infected CD4+ T cells. Intriguingly, the RT-PCR results showed that fungi treatment induced IFN-γ, IL-17A, and TNF-α expression in CD4+ T cells isolated from healthy controls (**Figures 3A–C**). In line with this, the expression of all three cytokines was significantly up-regulated in CD4+ T cells isolated from peripheral blood of activated CD and UC patients compared with their control counterparts. Next, we measured three transcription factors, including T-bet, RORC, and Foxp3, that determine the direction of CD4+ T cells differentiation to Th1, Th17, and Treg, respectively. The results showed that T-bet and RORC expression sharply increased upon fungi infection (**Figures 3D–F**). Consistently, the up-regulation of T-bet and RORC was much higher in IBD CD4+ T cells than in healthy control subjects.

However, Foxp3 expression remained unchanged in CD4+ T cells derived from healthy donors and IBD patients. These results indicate that fungi infection promotes CD4+ T cell differentiation into Th1 and Th17 but not T reg cell. To further confirm this, we measured the protein level of IFN-γ, IL-17A, and TNF-α under fungi infection in both healthy donors and IBD patients. The supernatant of CD4+ T cells culture medium was collected, followed by ELISA. The results indicated that protein expression levels of the inflammation cytokines IFN-γ, IL-17A, and TNF-α were significantly up-regulated in culture supernatants of fungi-infected CD4+ T cells compared with control CD4+ T cells (**Figures 3G–I**). Finally, colon specimens derived from IBD patients treated with terbinafine or those not treated with terbinafine were collected following IHC staining assays to detect IFN-γ, IL-17A, and TNF-α. As expected, IFN-γ, IL-17A, and TNF-α expression in the fungi-depleted colon sections were markedly reduced compared to those not treated with terbinafine (**Figure 3J**). These results suggest that fungi infection in CD4+ T cells enhances CD4+ T cell pro-inflammatory response in IBD patients.

### Fungi enhance glutaminolysis in CD4+ T cells

**Figure 4:**
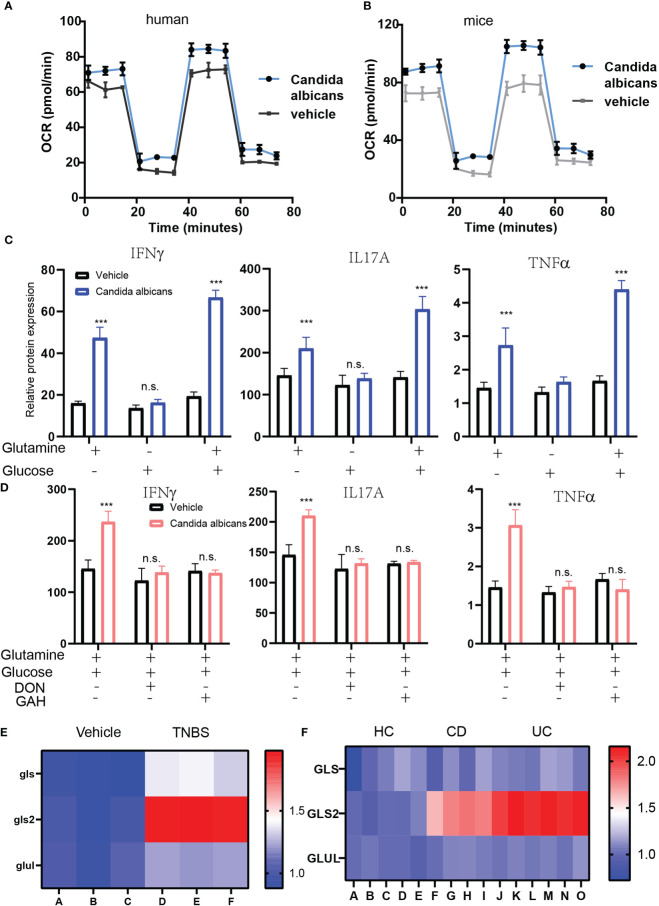
Enhanced glutaminolysis mediated by fungi infection is required for inflammation cytokines induction. **(A, B)**. The OCR level of CD4+ T cells isolated form human IBD **(A)** and mice **(B)** IBD that infected with fungi. **(C)**. The cytokines production of CD4+ T cells that supplemented with either 2 mM glutamine, 10 mM glucose or both. **(D)** The cytokines production of CD4+ T cells that blockage glutaminolysis with DON or GAH. **(E, F)**. The heat map of glutaminolysis genes expression in the fungi infected CD 4+ T cells. ***P < 0.001 and n.s., no significance P > 0.05.

Further, we aim to investigate the mechanism through which fungi promote CD4+ T cell pro-inflammatory immune responses in IBD patients. T cells enhance glucose and glutamine metabolism to elevate the level of mitochondrial OXPHOS for cytokine production. To investigate whether OXPHOS is involved in CD4+ T cell response under fungi infection, we detected OXPHOS levels in both fungi-infected and control humans as well as murine CD4 T cells by measuring OCR levels using seahorse analysis. As expected, fungi infection significantly induced the up-regulation of OCR levels in human CD4 T cells. Consistently, this enhanced OXPHOS was also observed in murine CD4 T cells, showing that OXPHOS increased by a maximum of about 40% (**Figures 4A, B**). Moreover, we found that suppressing OXPHOS with FCCP could block cytokine production induced by fungi infection.

Previous studies reported that glutamine metabolism plays a critical role in regulating the differentiation of T cells into different T helper subtypes. To assess whether glucose or glutamine mediate enhanced OXPHOS, we measured the production of inflammatory cytokines by CD4+ T cells under glucose or glutamine-deficiency culture conditions. The results showed that the glucose-free culture medium had almost no effect on IFN-γ production. However, the glutamine-free culture medium could reverse cytokine production by CD4+ T cells induced by fungi infection(**Figure 4C**). Moreover, IFN-γ production was highest in both glutamine and glucose-supplemented culture media. To further confirm that glutaminolysis in the CD4+ T cells determines cytokines production, we further blocked glutaminolysis in CD4+ T cells using glutaminolysis inhibitors, 6-Diazo-5-oxo-L-norleucine (DON) or L-Glutamic acid γ-monohydroxamate (GAH) (**Figure 4D**). The two inhibitors reversed the up-regulated cytokines production. Finally, we detected the glutamine metabolism enzymes GLS, GLS2, and GLUL mRNA expression under fungi infection in murine CD4+ T cells and CD4+ T cells obtained from CD and UC patients. The Q-PCR results showed that the expression of GLS2, which converts glutamine to glutamate, was markedly enhanced in activated CD4+ T cells (**Figures 4E, F**). In summary, fungi infection enhanced glutaminolysis in CD4+ T cells to support the production of cytokines.

### Fungi activate dectin-1-Syk- NF-κB to enhance the glutaminolysis in CD4+ T cells

**Figure 5:**
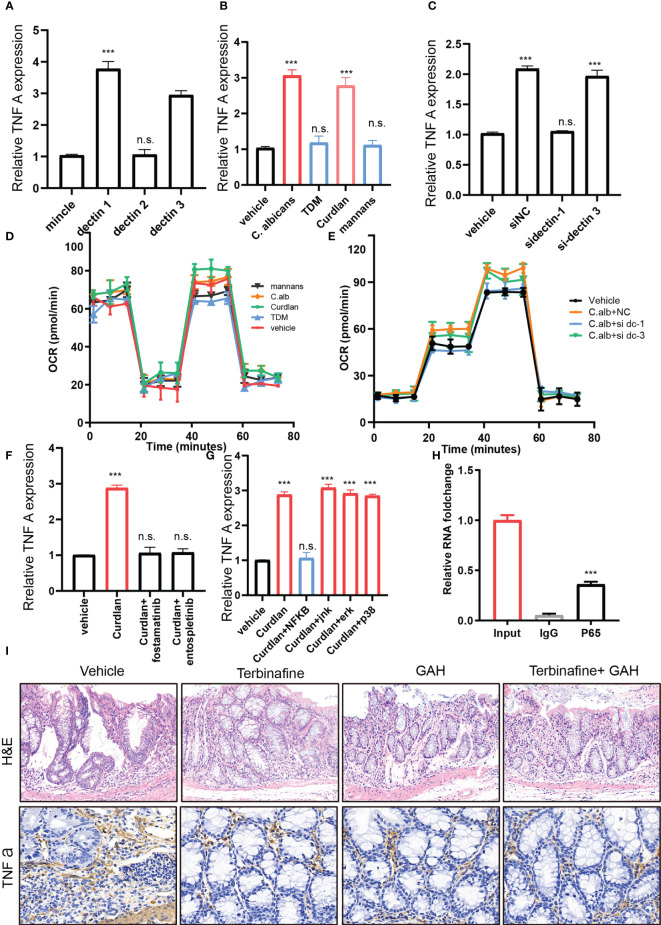
Fungi activates dectin-1-Syk- NF-κB aixs to enhancing glutaminolysis in CD4+ T cells. **(A)**. The relative mRNA expression of fungi recognize receptor expression in the IBD CD 4+ T cells. **(B)**. The TNFα expression under fungi infection or plus with mincle inhibitor TDM, dectin1 receptor Curdlan or dectin2/3 receptor mannans. **(C)**. The TNFa expression after dectin receptor knockdown under fungi infection. **(D, E)**. The OCR level of CD4 + T cells under C-type lectin receptors agonists **(D)** or C-type lectin receptors agonists knockdown. **(F)**. The TNFa expression under Curdian or Curdian puls SyK inhibitors. **(G)** The TNFA expression under Curdian or Curdian puls signaling pathway inhibitors. **(H)** The CHIP-PCR of TNFA with IgG or P65 antibody. **(I)** The H&E and IHC staining of colon sections derived from vehicle, terbinafine, GAH and terbinafine plus GAH treated mice. ***P < 0.001 and n.s., no significance P > 0.05.

In addition, we investigated the mechanism through which fungi enhance glutaminolysis in CD4+ T cells. Firstly, to investigate receptors that mediate fungi sensing in CD4+ T cells, we evaluated the expression of common receptors for fungi in the CD4+ T cells, including Mincle, Dectin-1, Dectin-2, and Dectin-3 ligands. The real-time PCR results indicate that Mincle and Dectin-2 were expressed on CD4+ T cells. To assess which receptor exerts the function, siRNA was introduced to knock down Mincle and Dectin-2 in CD4 T cells. The results showed that suppressed Mincle, but not Dectin-2, expression reduced glutaminolysis in CD4+ T cells. To further confirm this, trehalose-6,6-dimycolate (TDM) and α-mannans, and Mincle and Dectin-2/3 ligands were applied to treat the CD4+ T cells, respectively. We found that stimulation with α-mannans failed to induce glutaminolysis (**Figures 5A–D**). However, TDM stimulation significantly induced glutaminolysis (**Figures 5A,B**), and the deletion of Mincle in BMDCs completely blocked TDM-induced glutaminolysis (**Figures 5E, F**). Moreover, treating BMDCs or human peripheral blood mononuclear cells (PBMCs) with Syk inhibitor significantly impaired glutaminolysis in CD4+ T cells.

Previous studies have shown that Syk signaling could activate the NF-κB pathway. To reveal whether NF-κB is involved in this process, CD4+ T cells derived from human and mice IBD colon were treated with NF-κB inhibitors (**Figures 5G–I**). Consistently, NF-κB blockage significantly suppressed the glutaminolysis and glutamine metabolism genes expression. These findings suggested that fungi enhance glutaminolysis in CD4+ T cells by activating the dectin-1-Syk- NF-κB signaling pathway.

## Discussion

Gut microbiota alteration has been associated with the pathogenesis of multiple diseases, including cancer (York, 2018; Fernandes et al., 2022), Alzheimer’s disease (Pan et al., 2022), diabetes (Yuan et al., 2022), and chronic inflammatory diseases, especially IBD (Manichanh et al., 2012; Hov and Karlsen, 2022). However, studies have mostly focused on gut bacteria. Recently, the roles of fungi gradually attracted researchers’ attention (Iliev, 2022). However, how fungi interacted with immune cells in the pathogenesis of IBD is uncertain. This study found that intestinal fungi regulated immune responses in IBD, and terbinafine-mediated depletion of fungi ameliorated the development of IBD. Furthermore, fungi infection greatly enhanced OXPHOS by inducing glutaminolysis. Mechanistically, we found that fungi could activate the dectin-1-Syk- NF-κB signaling pathway to promote the expression of key enzymes. In summary, our findings reveal that fungi dysbiosis in the human gut could be a promising therapeutic target for IBD.

Fungi alteration and function in the development of IBD were recently acknowledged. *Candida albicans* was reported to be significantly increased in IBD, while the ratio of *Saccharomyces cerevisiae* markedly decreased (Sokol et al., 2017). However, the roles of fungi in the intestinal microenvironment and immune homeostasis remain unclear. Leonardi et al. reported that *D. hansenii* infection triggers CX3CR1+ mononuclear cells, enhancing the release of CCL5 (Leonardi et al., 2018). Our data showed that in IBD, *Candida albicans* can increase pro-inflammatory cytokines like IFN-γ, IL17A, and TNF-α production, further broadening the understanding of fungi roles in intestinal immune homeostasis.

Metabolism reprogramming has been reported to play an important function in regulating inflammation (O’Neill et al., 2016). Glycolysis is necessary for the lipopolysaccharide-mediated activation of macrophages and dendritic cells (Krawczyk et al., 2010; Rodriguez-Prados et al., 2010). In addition, carbohydrate kinase-like protein (CARKL) was found to limit macrophage switch into the M1 phenotype by suppressing the pentose phosphate pathway (Tannahill et al., 2013). On the contrary, fatty acid oxidation metabolism was triggered by PGC1 signaling to support macrophage differentiation into M2 macrophages (Vats et al., 2006). As for B cells, Jiang et al. reported that enhanced glutaminolysis is necessary for IL10 expression in allergic rhinitis (Liu et al., 2022). Similarly, this study showed fungi infection enhances glutaminolysis in CD4+ T cells to support the production of pro-inflammatory cytokines, resulting in IBD progression. Moreover, our results showed that blocking glutaminolysis in the CD4+ T cells could inhibit the expression of inflammation-related genes. Meanwhile, Stephanie et al. reported that transcription factor Foxo3a suppressed glutaminase expression, inhibiting glutaminolysis and hampering colitis induced by IL10 knockout. Collectively, glutamine metabolism is necessary for CD4+ T cell-mediated IBD development. Additionally, as for how glutaminolysis enhancement regulates the CD4+ T cells differention need more efforts in the further. On the other side, screening potential drugs that targeting glutaminolysis in CD4+ T cells would be a promising strategy for IBD therapy.

Immune cells recognize pathogens through pattern-recognition receptors (PRP) (Hardison and Brown, 2012), and C-type lectin receptors are the major PRP for fungi recognition. C-type lectin receptor family contains four members, including Mincle, dectin-1, dectin-2, and dectin-3 (Brown et al., 2018). Our study showed that dectin-1 is the receptor to recognize fungi in CD 4+ T cells, and dectin-1 depletion can prevent inflammation-related cytokine production. Consistent with our study, Sonja et al. reported that human dendritic cells sense fungi *via* dectin-1, resulting in interferon β production (Gringhuis et al., 2022). Moreover, MDSCs in CRC tumor microenvironments also recognized fungi through dectin-1 receptor (Malik et al., 2018). However, BMDCs was reported to sense the fungi through dectin-2/3 receptor, resulting in IL-10 production regulation (Duan et al., 2021). In this study, we also deciphered the signaling pathway that enhances gene expression of glutamine metabolism. The downstream signaling of dectin-1 is numerous, including MAPK-p38, ERK, NF-κB, and JNK signaling. Our data showed that only NF-κB inhibitor could reverse inflammatory cytokine production induced by fungi administration. In addition, the CHIP-PCR results indicated that P65 could trans-activate GLS2 expression in CD4+ T cells.

In summary, in this study, we found fungi dysbiosis in humans and mice with IBD, and terbinafine-mediated fungi depletion could delay the progression of IBD. Fungi infection greatly enhances oxidative phosphorylation (OXPHOS) by enhancing glutaminolysis. Mechanistically, we found that fungi could activate the dectin-1-Syk- NF-κB signaling pathway to promote the expression of key enzymes. Our findings reveal that disturbance of fungal interaction in the human gut could be a promising therapeutic target for inflammatory bowel disease.

## Data availability statement

The raw data supporting the conclusions of this article will be made available by the authors, without undue reservation.

## Ethics statement

The studies involving human participants were reviewed and approved by Shanghai Jiaotong University School of Medicine, Renji Hospital Ethics Committee. The patients/participants provided their written informed consent to participate in this study.

The animal study was reviewed and approved by Shanghai Jiaotong University.

## Author contributions

MY, HD and SG conducted experiments and collected the data; YL, HPL, YM, HL performed experiments and provided intellectual discussion. XL and MZ designed the experiments. MY, HD and SG wrote the manuscript. All authors contributed to the article and approved the submitted version.
