# Supplementary material for: Fungal dysbiosis facilitates inflammatory bowel disease by enhancing CD4+ T cell glutaminolysis
Source: Front Cell Infect Microbiol. 2023 Apr 14;13:1140757. doi: 10.3389/fcimb.2023.1140757 (PMC10140311; doi:10.3389/fcimb.2023.1140757)
Supplement: Supplementary file 1 [file DataSheet_1.docx]

Supplementary figure1


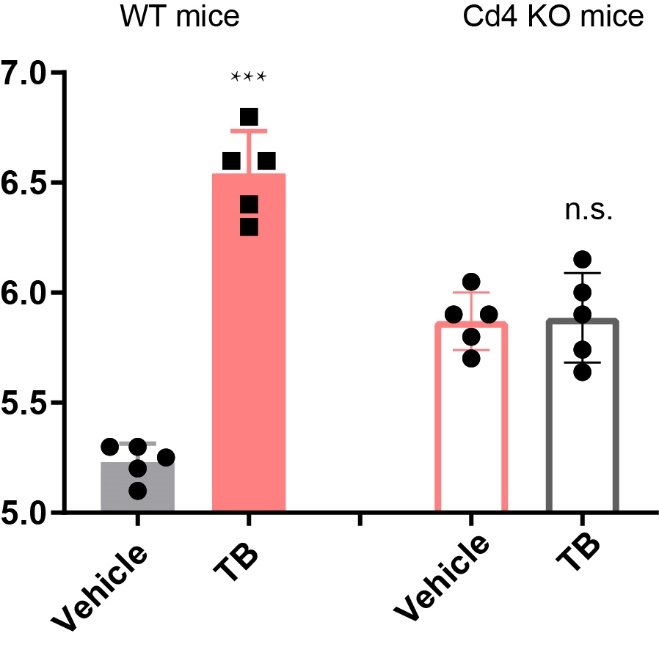


Supplementary figure1

Colon length of mice IBD with administration of terbinafine in WT or *Cd4* knock out mice
